# The Brain from Within

**DOI:** 10.3389/fnhum.2016.00265

**Published:** 2016-06-08

**Authors:** Umberto di Porzio

**Affiliations:** Institute of Genetics and Biophysics “A. Buzzati-Traverso”, Consiglio Nazionale delle Ricerche (CNR)Naples, Italy

**Keywords:** fMRI, neurofeedback, neuroimaging, neuroplasticity, rtfMRI, ethical issues, ASL, DTI

## Abstract

Functional magnetic resonance imaging (fMRI) provides a powerful way to visualize brain functions and observe brain activity in response to tasks or thoughts. It allows displaying brain damages that can be quantified and linked to neurobehavioral deficits. fMRI can potentially draw a new cartography of brain functional areas, allow us to understand aspects of brain function evolution or even breach the wall into cognition and consciousness. However, fMRI is not deprived of pitfalls, such as limitation in spatial resolution, poor reproducibility, different time scales of fMRI measurements and neuron action potentials, low statistical values. Thus, caution is needed in the assessment of fMRI results and conclusions. Additional diagnostic techniques based on MRI such as arterial spin labeling (ASL) and the measurement of diffusion tensor imaging (DTI) provide new tools to assess normal brain development or disruption of anatomical networks in diseases. A cutting edge of recent research uses fMRI techniques to establish a “map” of neural connections in the brain, or “connectome”. It will help to develop a map of neural connections and thus understand the operation of the network. New applications combining fMRI and real time visualization of one’s own brain activity (rtfMRI) could empower individuals to modify brain response and thus could enable researchers or institutions to intervene in the modification of an individual behavior. The latter in particular, as well as the concern about the confidentiality and storage of sensitive information or fMRI and lie detectors forensic use, raises new ethical questions.

## Introduction

In the last decades, extraordinary advances in image processing and quantification have provided new anatomical identification and function analysis of the human body, especially the brain. Imaging also provides an excellent display of structural brain damage that can be quantified and linked to neurobehavioral deficits. High-throughput nuclear magnetic resonance (NMR) and a number of its variations are already largely used in neuropathology.

**Figure 1:**
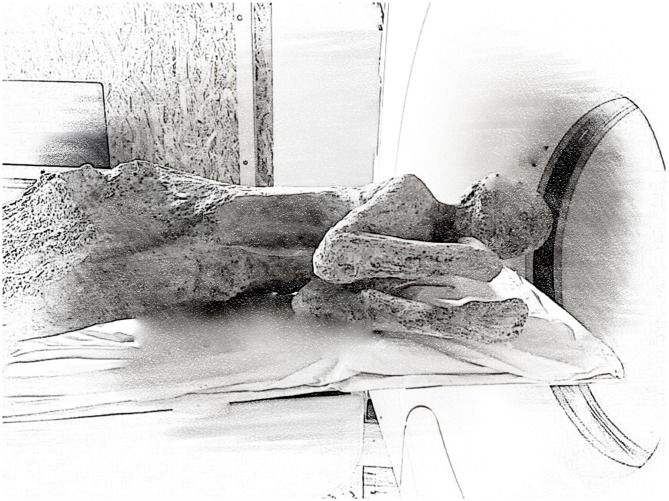
**Nuclear magnetic resonance (NMR) instrument used to analyze a 79 Alzheimer’s disease (AD) Pompeii’s eruption victim.** MRIs may work with magnets from 1.5 to 7 Tesla. Here is a sketch of the typical instrument used to scan the body of a Pompeii eruption victim, which was encased in solidified ash. (Modified from http://www.nationalgeographic.com/).

NMR makes it possible to look inside the brains noninvasively, but also to analyze the body of people died thousands of years ago (Figure 1). Based on the fact that cerebral blood flow and neuronal activation are coupled, functional NMR (fNMR) delivers instantaneous representation of brain activity and can locate areas of the brain correlated to specific behavior or cognitive functions. Thus, it allows to observe the brain while thinking, feeling and acting, and analyze brain responses to pharmacological treatments.

NMR method was developed over the last 20 years by researchers that were the recipient of seven Nobel Prize. In 1946, American physicists Felix Bloch and Edward Purcell, studying protons, discovered the phenomenon of resonance and in 1952 received the Nobel Prize for Physics. The Nobel Prize in Physiology or Medicine 2003 was awarded to Paul Lauterbur for his discovery that applied, magnetic field gradients could generate images (1973) and to Sir Peter Mansfield, who further perfected the technique by introducing changes in the magnetic fields, thus achieving more detailed images. In 1979, Cormack and Hounsfield received the Nobel Prize in Physiology or Medicine for the development of computer-assisted tomography**.** The 1991 Nobel in Chemistry was awarded to Richard R. Ernst for the development of NMR spectroscopy and Kurt Wüthrich in 2002 for NMR studies of structure and function of biological macromolecules.

## History of Neuroimaging

The first functional magnetic resonance imaging (fMRI) machine was built by Ogawa et al. (1990) at Bell Labs, Belliveau et al. (1991) at Massachusetts General Hospital mapped human visual cortex and Kwong et al. (1992) developed a method for visualizing and measuring blood flow and oxygen metabolism. The signals for the formation of NMR images are hydrogen protons in water, which revolve around an axis (have a spin) generating a microscopic magnetic field. Normally the atomic nuclei are randomly oriented but under the influence of a magnetic field they align with the field direction; the stronger, the field, the greater, the degree of alignment. The resonance signals of the “magnetizable” molecules are measured with the help of magnetic fields and radio waves. Radio waves make molecules oscillate in tissues. These oscillating molecules emit signals, i.e., they resonate.

The signals are recorded and further analyzed by computers. The power of the unit and thus its capacity of resolution, that is, capturing distinct signals in time and space, is defined by the power of the magnetic fields (today common fMRI reach up to 3 Tesla although high resolution 7–9 T instrument are available) and also by the number of sensors capable of capturing variations of magnetic signals (scanners of the latest generation have up to 200 antennas of detection). The tesla (T) is the unit of measurement of the magnetic field, equivalent to 10,000 Gauss.

**Figure 2:**
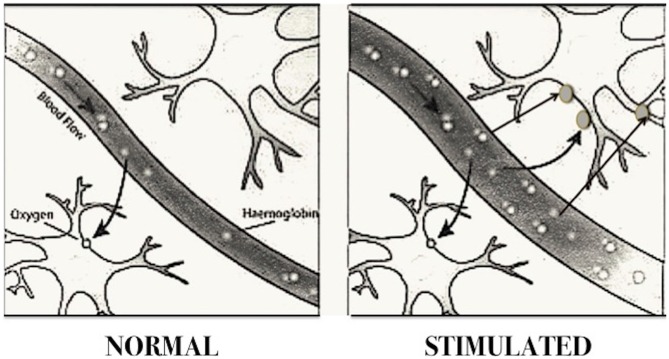
**Blood oxygen level dependent (BOLD) imaging.** The diagram shows how cerebral blood flow increases according to neuronal activity. Brain areas activated in specific tasks require a greater supply of oxygen by augmenting blood flow, which provides greater amount of oxyhemoglobin. Since the latter is diamagnetic, that is rejected by the magnetic field, while the deoxygenated is paramagnetic, they behave differently in a magnetic field. The instrument sensors are able to pick up the electromagnetic waves sent back by the two forms of hemoglobin, and reconstruct a computerized image of the cerebral localization of hemoglobin molecules. The regions with increased oxyhemoglobin correspond to the areas of increased neuronal activity. This method is called “magnetic resonance images dependent” (BOLD). (Modified from Dr. *Stuart Clare, FMRIB, University of Oxford, UK)*.

Specific brain areas are recruited when animals or people perform tasks (such as moving a hand, reading words or looking at objects). The neurons recruited in the task require more oxygen. fMRI measures the blood flow in the brain as changes in the magnetic properties, owing to oxygenation of the blood (the blood oxygen level dependent (BOLD) signal, see Figure 2), by measuring the difference in magnetism between oxygenated and deoxygenated hemoglobin. In fact, hemoglobin is diamagnetic when oxygenated (oxyhemoglobin), and therefore rejected by the magnetic field, but becomes paramagnetic when deoxygenated (deoxyhemoglobin), i.e., attracted by the magnetic field, One of the problems encountered in studies using fMRI is that fMRI provides qualitative information on brain function, but not quantitative, therefore many scholars actively seek to develop new methods for the measurement of oxygen metabolism, and new strategies to increase the resolution of the captured images.

Angelo Mosso, an Italian scientist, at the end of the 19th century was the first person to intuit that brain activity could be inferred by measuring changes in blood flow (Sandrone et al., 2014). Towards the end of the 19th century connection between energy metabolism and blood flow in the brain was demonstrated (Roy and Sherrington, 1890). Kety and Schmidt (1948) demonstrated that the brain itself regulates blood flow in the brain, i.e., when neurons use more oxygen, chemical signals cause dilation of the neighboring blood vessels.

Sophisticated instruments are now available to investigate the connectivity of the brain with integration of function and anatomy. fMRI is also a powerful means of investigation to understand the basics of how this complex organ functions and its peculiarities in different species and their evolution. For instance, a region of the human brain sensitive to human voice has been identified and scientists asked if this region is associated with linguistic processing and is unique to humans. To answer this question, macaques (*Macaca mulatta*) were submitted to fMRI scans. The results show that a region in the brain responds to species-specific vocalization, in contrast to other vocalization, showing sensitivity to the “voice” of the species that is the ability to identify the voice of individuals of the same species. These results support the idea that the anterior temporal regions of the primate brain are able to recognize communication signals from conspecifics (Petkov et al., 2008, 2009). Thus have established a functional relationship between the brain region related to the human voice and that of the macaque.

## Future Developments

Recently a method that measures blood flow changes directly, the arterial spin labeling (ASL) has been developed. ASL and BOLD imaging used together could provide a more quantitative assay of brain function, including assessment of oxygen metabolism changes during the development of neurological diseases. In this regard, ASL can be regarded as a promising tool for early diagnosis and for the description of the progression of Alzheimer’s disease (AD; Wierenga et al., 2014). Several studies in which ASL was used, also showed that blood flow decreases as AD develops, showing hypoperfusion both globally and in selective regions. The study also showed considerable overlap between brain regions associated with the disease and those associated with the state of risk (Alsop et al., 2010). Thus, this new fMRI technical approach supports the hypothesis that AD is initially linked to vascular disease, rather than ascribable only to the amyloid hypothesis.

An additional new diagnostic technique of MRI based on the measurement of diffusion tensor imaging (DTI), proved to be a valuable tool for the *in vivo* study of white matter and neurological disease (Alexander et al., 2007). The white matter is rather fibrous and water diffuses along the direction of the fibers, making it possible to trace the structure and orientation of the neural fiber bundles (Fiber tracking or Tractography), thus the vectorial velocity of the information flow. It can therefore allow the detection and characterization of development and organization of neural connectivity as well as disruption of brain networks in diseases (Weiner et al., 2015; Li et al., 2016; Sun et al., 2016).

The use of fMRI to establish a “map” of neural connections in the brain, or “connectome”, is at the cutting edge of recent research. In other words, to understand the operation of a network, one must know its elements and their interconnections. Thus it is necessary to develop a map of neural connections, of brain cabling. These studies will increase our understanding of how the functional state of the brain emerges from its structural substrate, and provide mechanistic information on how the brain functions are impaired if this structural substrate is “interrupted or altered” (Sporns et al., 2005). Soon it should be possible to establish a type of map of the connections between brain areas of the healthy human brain, and to understand how information is processed. Such knowledge should also help in the development of new drugs for neurodegenerative diseases and pain. However, it appears difficult for now to translate neuroimaging advances into diagnostic tools for psychiatry (Stephan et al., 2015).

New tools as the Allen Brain atlas, attempt to integrate extensive gene expression data, genomics and functional neuroanatomy, coupled to fMRI, to understand which group of genes is expressed in a particular neuron in a given location and in response to certain stimuli. For example, putting together information on gene expression in a specific area of the brain has shown that different sections (or brain slices) of the cortex are defined by completely different sets of genes. This approach supports the view that the brain does not work as a Turing machine, i.e., it does not work as a supercomputer that is based on the calculation and processing of information, but selects groups of neurons, whose molecular and functional characteristics can be modified, as hypothesized by Edelman (1993) in his Theory of Neural Darwinism.

Neuroimaging approaches also show that some definitions of anatomical areas, so far completely accepted and considered valid, represent old maps that are not only obsolete, but also even misleading. As an example, until recently the hippocampus was divided into four distinct areas. Current data indicate that a single area of the hippocampus can be divided into at least nine distinct regions, each with its own unique gene expression.

It is now possible to associate other information on the functioning neurons obtained with additional techniques, such as measuring the electrical activity of single neurons or measuring the tiny magnetic fields generated by brain activity with Magnetoencelphalography (MEG). Altogether, this information will improve our possibility to understand how healthy and diseased brains differ. Since the individual genetic variability among healthy humans is extremely high, only information collected on many samples and statistically validated can be taken into consideration.

Surprisingly one of the fields in which fMRI has offered a major contribution to our understanding are the studies of music and emotion as well as musical learning, performing and listening (Koelsch, 2014). New approaches are also using functional connectivity MRI with the use of a computer, which has been programmed to compare large numbers of brain scan data and build a map that can be used as a reference to compare single brain scans (Dosenbach et al., 2010).

It is interesting to note that fMRI revealed that in principle, the brain could respond to a so-called placebo, a simulated medical intervention or sham treatment, in the same way with which it responds to the activity of a therapeutic treatment. In recent experiments patients receiving the placebo showed responses similar to those of patients who received the treatment for pain and analgesia. Using fMRI, it has been seen that also with the placebo, the analgesia was linked to decreased brain activity in brain regions sensitive to pain (insula, thalamus, and anterior cingulate cortex; Wager and Atlas, 2015). This was one of the first studies to show that the brain can regulate its own response in anticipation of specific events.

A new approach allows healthy or diseased subjects to learn how to observe the responses of their own brain and monitor its activation. That is, through fMRI training participants can control their own brain activity and specific brain networks by neurofeedback. This method, called real-time fMRI (rtfMRI) could potentially lead to changes in behavior and cognitive processes in healthy subjects and patients, a form of cerebral rehabilitation based on the use-dependent plasticity, with potentially long-lasting changes both in the normal and diseased states. A use that could also be modified by medications or drugs, to change memory, responses, and/or the character at will of the subject or of the operators (Brühl, 2015). Thus, rtfMRI shows that humans can adjust the hemodynamic in specific regions of the brain, changing behavioral response. Methods to better determine the nature of the dynamics of the functional interactions between different brain regions and plasticity through the formation of self-regulation are still under development. In essence, it would be possible to achieve a reorganization of the brain during learning to control local cerebral activity. It is still unclear how learning and the real-time view of the activation of specific brain areas in their own brain may allow individuals to modify responses of these areas. These techniques, albeit still relatively limited, have already been applied to studies on sleep and sleep deprivation (Muto et al., 2012; Mascetti et al., 2013), chronic pain (Guan et al., 2015), control of adverse stimuli (Paret et al., 2015), obsessive-compulsive disorder (Buyukturkoglu et al., 2015), depression (Linden et al., 2012) and schizophrenia (Ruiz et al., 2013). For an extensive list, see Table 1 in Brühl (2015).

To date, many studies show good acquisition of control inside the scanner, but the evidence for persistent behavioral changes in real life after training is limited (Stoeckel et al., 2014).

Although some concern and apprehension are raised by rtfRMI hypothetical misuse in altering people’s brain activity or free will, this methods offers the opportunity to further our understanding of how the brain works, by facilitating specific changes in brain function through self-directed neuroplasticity. Indeed we know that the brain is capable of changing its function in response to environment, thinking, emotions, behavior, as well as injury. The applications can produce changes in cognition, experience or behavior by optimization of brain plasticity and pushes the limits of our potential for self-directed healing and adjustment. Furthermore, fMRI and rtfMRI can help optimize CNS drug discovery for CNS disorders.

## Flaws

Although fMRI undoubtedly is an advanced technology, able to identify areas of the brain that respond to a given stimulus, movement, action, and perhaps thought, the methodology is not without limitations. The major and well-known flaws are related to spatial and temporal resolution. Millions of neurons reside in the smallest cube-sized area of gray matter and neural signals are transmitted much quicker than those from blood. The technique does not measure neuronal activity directly and cannot see details such as how many neurons are firing, or whether firing in one region amplifies or dampens activity in neighboring areas. It can identify large, active brain areas, but might miss clumps of inactive neurons within it or small islands of active neurons in inactive areas.

A major unresolved issue is how to translate in the slow oxygenation of the blood, 3–6 s, the rapid time (ms) required for a neuron to generate action potentials, i.e., activity.

A further element of confusion on the interpretation of results from fMRI is the low average statistical *P* values that generate some uncertainty about reproducibility and reliability. In fact, the brains of different individuals are anatomically different, and the brain of a single individual changes with age or disease. Experiments with repetition of the same fMRI in the same individual for a specific task do not always “turn on” the same brain areas (Bennett and Miller, 2010; Button et al., 2013).

## Legal, Social and Ethical Issues

fMRI studies are gradually raising new ethical issues, as investigations of neuronal models associated with decision-making or memory recovery, personality traits, etc. multiply. Just in the last 5 years, NMR studies in PubMed number about 50,000, one third related to brain spectroscopy.

The aim to correlate the different mental states and processes with neuronal activity in defined brain areas, might one day be used to decode mental activity, could find uses unrelated to medicine or biology, potential misuse aimed at gathering information that is usually legally prohibited. That is, employed to detect personality traits and mental capacities to identify tendencies and capacities, trends of character and ability of individuals or applicants by insurance companies, employers, schools (Haynes and Rees, 2006; Haynes et al., 2007). These cases are still largely hypothetical.

Indeed, fMRI research advancements elicit major, largely unfounded, concerns amongst civil libertarians, who worry that it could become a threat to individual privacy. Barry Steinhardt, director of the American Civil Liberties Union’s Technology and Liberty Project, declared “They are going to read people’s thoughts‥. little attention has been paid to the potential misuse of fMRI and the devastating impact it would have on our civil liberties.” (in Adler, 2007), Moreover the issue of confidentiality of personal information collected through these methods of investigation and their storage raises alarm. These issues regard ethical rules and legal and social guarantees, professional ethics and democratic warranties about individual freedom and privacy control. In addition, in this case it regards also the intimacy of one’s thoughts (Garnett et al., 2011).

Equally, lie detector fMRIs are being developed based on the ability to distinguish patterns of brain activation that correspond to a deliberate attempt to mislead, from patterns that correspond to truth. These changes largely consist of the activation of ventrolateral prefrontal cortex in individuals who lie with respect to individuals who tell the truth. But lies do not represent a homogeneous category of behavior. There is deception aimed at personal gain or instead to save the feelings of another person, lies we say to ourselves and lies that intend to deceive by affirmation or by omission of information. Each of these can have different neural correlates, including the degree of reiteration or emotion associated with the lies. It is understandable why attempts to apply these techniques in legal cases arouse major controversy (Farah et al., 2014).

Nevertheless, the possibility to monitor the functions of the brain and to modify its responses opens important avenues for diagnosis, to direct therapeutic intervention, to assess the mechanism and efficacy of treatments. It must be kept in mind that constraints do exist, and that there are problems of reproducibility of data, of intrinsic variations amongst individuals and the conditions (normal or pathological, young or old) of individual brains, therefore data should evaluated with stringent statistical methods.

In conclusion, alongside important research potential and ability of improving therapeutic interventions, fMRI raises new ethical issues and the need to preserve sensitive information and equally, the privacy of the mind and thinking.

## Author Contributions

UdiP is the only author. The author confirms being the sole contributor of this work and approved it for publication.

## Conflict of Interest Statement

The author declares that the research was conducted in the absence of any commercial or financial relationships that could be construed as a potential conflict of interest.

## References

[B1] Adler K. (2007). The Lie Detectors: The History of An American Obsession. New York, NY: Free Press.

[B2] Alexander A. L. Lee J. E. Lazar M. Field A. S. (2007). Diffusion tensor imaging of the brain. Neurotherapeutics 4, 316–329. 10.1016/j.nurt.2007.05.011 17599699 PMC2041910

[B3] Alsop D. C. Dai W. Grossman M. Detre J. I. (2010). Arterial spin labeling blood flow MRI: its role in the early characterization of Alzheimer’s disease. J. Alzheimers Dis. 20, 871–880. 10.3233/JAD-2010-091699 20413865 PMC3643892

[B4] Belliveau J. W. Kennedy D. N. Jr. McKinstry R. C. Buchbinder B. R. Weisskoff R. M. Cohen M. S.. (1991). Functional mapping of the human visual cortex by magnetic resonance imaging. Science 254, 716–719. 10.1126/science.1948051 1948051

[B5] Bennett C. M. Miller M. B. (2010). How reliable are the results from functional magnetic resonance imaging? Ann. N Y Acad. Sci. 1191, 133–155. 10.1111/j.1749-6632.2010.05446.x 20392279

[B6] Brühl A. B. (2015). Making sense of real-time functional magnetic resonance imaging. (rtfMRI) and rtfMRI neurofeedback. Int. J. Neuropsychopharmacol. 18:pyv020. 10.1093/ijnp/pyv020 25716778 PMC4438554

[B7] Button K. S. Ioannidis J. P. Mokrysz C. Nosek B. A. Flint J. Robinson E. S.. (2013). Power failure: why small sample size undermines the reliability of neuroscience. Nat. Rev. Neurosci. 14, 365–376. 10.1038/nrn3475 23571845

[B8] Buyukturkoglu K. Roettgers H. Sommer J. Rana M. Dietzsch L. Arikan E. B.. (2015). Self-regulation of anterior insula with real-time fMRI and its behavioral effects in obsessive-compulsive disorder: a feasibility study. PLoS One 10:0135872. 10.1371/journal.pone.0145027 26301829 PMC4547706

[B9] Dosenbach N. U. Nardos B. Cohen A. L. Fair D. A. Power J. D. Church J. A.. (2010). Prediction of individual brain maturity using fMRI. Science 329, 1358–1361. 10.1126/science.1194144 20829489 PMC3135376

[B10] Edelman G. M. (1993). Neural darwinism: selection and reentrant signaling in higher brain function. Neuron 10, 115–125. 10.1016/0896-6273(93)90304-a 8094962

[B11] Farah M. J. Hutchinson J. B. Phelps E. A. Wagner A. D. (2014). Functional MRI-based lie detection: scientific and societal challenges. Nat. Rev. Neurosci. 15, 123–131. 10.1038/nrn3665 24588019

[B12] Garnett A. Whiteley L. Piwowar H. Rasmussen E. Illes J. (2011). Neuroethics and fMRI: mapping a fledgling relationship. PLoS One 6:18537. 10.1371/journal.pone.0018537 21526115 PMC3081297

[B13] Guan M. Ma L. Li L. Yan B. Zhao L. Tong L.. (2015). Self-regulation of brain activity in patients with postherpetic neuralgia: a double-blind randomized study using real-time FMRI neurofeedback. PLoS One 10:0123675. 10.1371/journal.pone.0123675 25848773 PMC4388697

[B14] Haynes J. D. Rees G. (2006). Decoding mental states from brain activity in humans. Nat. Rev. Neurosci. 7, 523–534. 10.1038/nrn1931 16791142

[B15] Haynes J. D. Sakai K. Rees G. Gilbert S. Frith C. Passingham R. E. (2007). Reading hidden intentions in the human brain. Curr. Biol. 17, 323–328. 10.1016/j.cub.2006.11.072 17291759

[B16] Kety S. S. Schmidt C. F. (1948). The nitrous oxide method for the quantitative determination of cerebral blood flow in man: theory, procedure and normal values. J. Clin. Invest. 27, 476–483. 10.1172/jci101994 16695568 PMC439518

[B17] Koelsch S. (2014). Brain correlates of music-evoked emotions. Nat. Rev. Neurosci. 15, 170–180. 10.1038/nrn3666 24552785

[B18] Kwong K. K. Belliveau J. W. Chesler D. A. Goldberg I. E. Weisskoff R. M. Poncelet B. P.. (1992). Dynamic magnetic resonance imaging of human brain activity during primary sensory stimulation. Proc. Natl. Acad. Sci. U S A 89, 5675–5679. 10.1073/pnas.89.12.5675 1608978 PMC49355

[B19] Li C. Huang B. Zhang R. Ma Q. Yang W. Wang L.. (2016). Impaired topological architecture of brain structural networks in idiopathic Parkinson’s disease: a DTI study. Brain Imaging Behav. [Epub ahead of print]. 10.1007/s11682-015-9501-6 26815739

[B20] Linden D. E. Habes I. Johnston S. J. Linden S. Tatineni R. Subramanian L.. (2012). Real-time self-regulation of emotion networks in patients with depression. PLoS One 7:e38115. 10.1371/journal.pone.0038115 22675513 PMC3366978

[B21] Mascetti L. Muto V. Matarazzo L. Foret A. Ziegler E. Albouy G.. (2013). The impact of visual perceptual learning on sleep and local slow-wave initiation. J. Neurosci 33, 3323–3331. 10.1523/JNEUROSCI.0763-12.2013 23426660 PMC6619511

[B22] Muto V. Shaffii-le Bourdiec A. Matarazzo L. Foret A. Mascetti L. Jaspar M.. (2012). Influence of acute sleep loss on the neural correlates of alerting, orientating and executive attention components. J. Sleep Res. 21, 648–658. 10.1111/j.1365-2869.2012.01020.x 22594455

[B23] Ogawa S. Lee T. M. Kay A. R. Tank D. M. (1990). Brain magnetic resonance imaging with contrast dependent on blood oxygenation. Proc. Natl. Acad. Sci. U S A 87, 9868–9872. 10.1073/pnas.87.24.9868 2124706 PMC55275

[B24] Paret C. Ruf M. Gerchen M. F. Kluetsch R. Demirakca T. Jungkunz M.. (2015). fMRI neurofeedback of amygdala response to aversive stimuli enhances prefrontal-limbic brain connectivity. Neuroimage 125, 182–188. 10.1016/j.neuroimage.2015.10.027 26481674

[B26] Petkov C. J. Kayser C. Steudel T. Whittingstall K. Augath M. Logothetis N. K. (2008). A voice region in the monkey brain. Nat. Neurosci. 11, 367–374. 10.1038/nn2043 18264095

[B25] Petkov C. I. Logothetis N. K. Obleser J. (2009). Where are the human speech and voice regions and do other animals have anything like them? Neuroscientist 15, 419–429. 10.1177/1073858408326430 19516047

[B27] Roy C. S. Sherrington C. S. (1890). On the regulation of the blood-supply of the brain. J. Physiol. 11, 85–158. 10.1113/jphysiol.1890.sp000321 16991945 PMC1514242

[B28] Ruiz S. Lee S. Soekadar S. R. Caria A. Veit R. Kircher T.. (2013). Acquired self-control of insula cortex modulates emotion recognition and brain network connectivity in schizophrenia. Hum. Brain Mapp. 34, 200–212. 10.1002/hbm.21427 22021045 PMC6869886

[B29] Sandrone S. Bacigaluppi M. Galloni M. R. Cappa S. F. Moro A. Catani M.. (2014). Weighing brain activity with the balance: angelo Mosso’s original manuscripts come to light. Brain 137, 621–633. 10.1093/brain/awt091 23687118

[B30] Sporns O. Tononi G. Kötter R. (2005). The human connectome: a structural description of the human brain. PLoS. Comput. Biol. 1:42. 10.1371/journal.pcbi.0010042 16201007 PMC1239902

[B31] Stephan K. E. Iglesias S. Heinzle J. Diaconescu A. O. (2015). Translational perspectives for computational neuroimaging. Neuron 87, 716–732. 10.1016/j.neuron.2015.07.008 26291157

[B32] Stoeckel L. E. Garrison K. A. Ghosh S. Wighton P. Hanlon C. A. Gilman J. M.. (2014). Optimizing real time fMRI neurofeedback for therapeutic discovery and development. Neuroimage Clin. 5, 245–255. 10.1016/j.nicl.2014.07.002 25161891 PMC4141981

[B33] Sun Y. Chen Y. Lee R. Bezerianos A. Collinson S. L. Sim K. (2016). Disruption of brain anatomical networks in schizophrenia: a longitudinal, diffusion tensor imaging based study. Schizophr. Res. 171, 149–157. 10.1016/j.schres.2016.01.025 26811255

[B34] Wager T. D. Atlas L. Y. (2015). The neuroscience of placebo effects: connecting context, learning and health. Nat. Rev. Neurosci. 16, 403–418. 10.1038/nrn3976 26087681 PMC6013051

[B35] Weiner M. W. Veitch D. P. Aisen P. S. Beckett L. A. Cairns N. J. Cedarbaum J.. (2015). Impact of the Alzheimer’s disease neuroimaging initiative, 2004 to 2014. Alzheimers Dement. 11, 865–884. 10.1016/j.jalz.2015.04.005 26194320 PMC4659407

[B36] Wierenga C. E. Hays C. C. Zlatar Z. Z. (2014). Cerebral blood flow measured by arterial spin labeling MRI as a preclinical marker of Alzheimer’s disease. J. Alzheimers Dis. 42, S411–S419. 10.3233/JAD-141467 25159672 PMC5279221

